# Acute Mastoiditis

**Published:** 1911-09-30

**Authors:** 


### OTOLOGY.

### ACUTE MASTOIDITIS.

In all cases of acute suppuration in the middle ear the lining membrane of the antrum doubtless participates to some extent in the inflammation. In fact, the tympanum forms only a part, and is in a sense a diverticulum, of the entire middle-ear cavity, which includes the antrum, aditus, attic, and Eustachian tube. It would therefore be surprising if an acute inflammation were limited to the tympanum and failed to affect the other parts of the cavity. However, it is only when a state of tension or pressure is set up by the retention of inflammatory products, or by the violence of the inflammation, that any symptoms occur which point to the involvement of the antrum or mastoid cells; the small size of the channel of communication between the antrum and the tympanum, which is called the aditus, renders this retention easy.

Acute suppuration may involve the antrum and all the mastoid cells, or it may be limited to a few cells or to the antrum alone; owing to the better drainage of the latter cavity, suppuration there sometimes subsides, while it persists in some of the mastoid cells. So also the acute inflammation in the tympanum may sometimes clear up, and in rare cases even the perforation of the membrane may heal, although pus remains pent up in the mastoid.

The cardinal symptoms are fever, headache, mastoid pain, and tenderness on pressure. The temperature is, according to its height, accompanied by the ordinary febrile symptoms. As a guide to the severity of the disease the degree of fever is of little value. In every case of acute suppuration in the middle ear fever is present, which subsides after perforation of the drum. If, however, the mastoid is involved, fever generally persists after perforation; but this is by no means always so, especially in influenzal cases, and in any case it is seldom high—rarely above 101° F.—and is not raised in proportion to the extent or severity of the mastoid disease. Pain also varies considerably; in acute otitis it is localised in the depths of the canal or beneath the tragus, but it is referred to the back of the ear when the mastoid process is involved. Tenderness is a constant symptom, and can always be elicited by digital pressure, made with gradually increasing firmness, over the antral region; the slightest degree of tenderness will be declared by an involuntary wincing. Tenderness of inflamed glands below the mastoid can hardly cause confusion if ordinary care be taken.

The membrana tympani is much inflamed and the landmarks obliterated. There may be marked bulging, but by the time that the case is declared as mastoiditis there is generally a perforation. If there is a perforation the discharge is usually profuse, and quickly reappears after removal owing to overflow from the antrum. A most important sign is a bulging of the deep part of the postero-superior wall of the meatus, due to œdema of the soft parts; this is the result of the spread of inflammation from the antrum to this, the point on the surface nearest to that cavity, and is pathognomonic of antral disease. In advanced cases the skin over the mastoid becomes reddened and then swollen, so that the auricle is pushed forward; the retro-auricular furrow is not obliterated, and this is a most important point of distinction from the somewhat similar swelling spreading from furunculosis of the external meatus. Later a subperiosteal abscess may form, which generally communicates by a fistula through the bone with the mastoid cells; and finally this may rupture and a sinus through the skin result.

As already mentioned, an acute inflammation of the tympanum may subside, leaving the disease active in the mastoid region; but even in such cases tenderness on pressure always remains, and is therefore a sign of the highest importance. The other reliable sign is the bulging of the meatal wall; if these two signs are taken at the proper valuation, mistakes in diagnosis should be very few. Occasionally the constitutional symptoms in severe cases may simulate a specific fever, especially typhoid; the mistake will seldom be made if the ears are examined in such doubtful cases, but a blood-count may also be of value, for the leucocytes are increased in inflammatory affections, but diminished in enteric fever and influenza.

When seen quite early, and if there are no severe symptoms, such as those of cerebral involvement, the case may be at first treated as one of acute otitis media. The most important point is paracentesis of the drum if there is as yet no perforation, or free enlargement of the existing perforation unless it is of ample size. Other details consist of leeches, syringing with hot antiseptics, the application of hot fomentations, rest in bed, and a brisk purge. These measures must not be persisted in; if there is no marked improvement within 24, or at latest 36 hours, the mastoid should be opened without hesitation. In these acute cases the radical operation should not be performed, unless the history shows that it is in reality an acute exacerbation in the course of chronic disease. The operation recommended is that which goes by the name of Schwartze; the antrum is opened with gouges from behind the ear, all the mastoid cells opened up, granulations curetted, any overhanging bone cut away so that a funnel-shaped cavity is formed, and any disease followed up even to exposing the dura mater or lateral sinus. Little harm results from the exposure of these structures, and certainly less than leaving them in contact with disease. It is important to ensure free communication between the antrum and tympanum by cautious curettage of the aditus. The cavity is then lightly and evenly packed with ribbon gauze and the greater part of the wound left open. After the first two or three days the packing is repeated daily until the discharge from the ear has completely ceased and the cavity filled up. If this operation is performed in good time the perforation in the drum may be expected to heal, and the restoration of the hearing to be practically complete.

